# Case Report: A rare case of thyroid metastasis from breast cancer

**DOI:** 10.3389/fonc.2025.1692891

**Published:** 2025-10-22

**Authors:** Xiaochuan Gao, Mengxin Li, Jinghui Hong, Yuheng Wu, Tong Fu, Dong Song

**Affiliations:** Department of Breast Surgery, General Surgery Center, First Hospital of Jilin University, Changchun, China

**Keywords:** breast cancer, metastatic breast carcinoma to the thyroid, diagnostic challenge, case, literature review

## Abstract

**Introduction:**

To analyze the clinicopathological features, diagnosis, and treatment strategies for metastatic breast carcinoma to the thyroid (MBCT) to enhance clinical awareness of this rare condition.

**Methods:**

Analysis of clinical data from one MBCT patient and literature review.

**Results:**

A 41-year-old female with left breast invasive ductal carcinoma (IDC) received neoadjuvant AC-T chemotherapy, breast-conserving surgery, radiotherapy, and endocrine therapy. At eight years post-diagnosis, thyroid lesions were detected. Total thyroidectomy with lymph node dissection confirmed MBCT pathologically. No progression was observed at 16 months post-thyroidectomy.

**Conclusions:**

MBCT is a rare clinical entity characterized by nonspecific clinical and radiological findings. Immunohistochemical (IHC) analysis is essential for a definitive diagnosis. In patients with a breast cancer history, MBCT should always be considered in the differential diagnosis of thyroid abnormalities.

## 1 Introduction

Breast cancer (BC) is the most frequently diagnosed malignancy among women worldwide and ranks second globally in overall incidence, surpassed only by lung cancer (1). BC exhibits distinct epidemiological features and marked heterogeneity (2). IDC is the predominant pathological subtype, accounting for approximately 75% of all invasive BC cases (3). Distant metastasis (DM) is the leading cause of death in BC patients. After the onset of DM, the median overall survival (mOS) is approximately 24 months, and the 5-year survival rate declines to below 20% (4). The most frequent sites of DM from BC include the bone (30-60%), lung (21-32%), liver (15-32%), and brain (4-10%) (5–7). Metastatic site distribution in BC exhibits considerable variation, and involvement of certain organs, such as the thyroid gland, small intestine (8), vagina (9), is extremely rare. Thyroid metastasis from BC is uncommon. Despite the thyroid gland’s abundant vascular supply, it rarely becomes a site of metastatic disease, accounting for less than 0.2% of fine-needle aspiration cytology (FNAC) examinations (10). According to a recent literature review, the most common non-thyroidal malignancies (NTM) metastasizing to the thyroid gland include renal cell carcinoma (48.1%), colorectal carcinoma (10.4%), lung cancer (8.3%), BC (7.8%), and mesenchymal tumors (4.0%) (11). Although BC is among the primary malignancies responsible for thyroid metastases (TM), metastatic BC to the thyroid remains clinically uncommon, given the high incidence of BC and the overall low prevalence of TM.

## 2 Case description

### 2.1 Clinical data and initial diagnosis

A 41-year-old woman presented to the Department of Breast Surgery in October 2015 with a four-month history of a left breast mass. Physical examination revealed symmetrical breasts. A firm mass measuring approximately 3 cm × 2 cm with ill-defined margins and an irregular shape was palpable in the upper quadrant of the left breast. No significant lymph node enlargement was palpable in the bilateral axillary or supraclavicular regions.

#### 2.1.1 Breast color doppler ultrasound

A hypoechoic mass measuring 23.7 mm × 17.5 mm with ill-defined margins and an irregular contour was observed in the upper outer quadrant of the left breast, near the 12 o’clock position. Color Doppler imaging revealed no significant vascularity within the lesion. No abnormal lymph nodes were detected in the bilateral axillae. The lesion was classified as BI-RADS Category 4B.

#### 2.1.2 Mammography

A high-density mass opacity measuring approximately 2.5 cm × 2.5 cm was visualized in the upper outer quadrant of the left breast. The mass demonstrated ill-defined margins but had a relatively regular shape. The right breast showed no detectable mass, and no abnormal lymph nodes were identified bilaterally.

#### 2.1.3 3.0T Breast MRI (non-contrast and dynamic contrast-enhanced imaging)

An irregular nodular lesion, approximately 2.1 cm × 1.4 cm, was identified in the upper outer quadrant of the left breast. The lesion appeared mildly hypointense on T1-weighted imaging (T1WI) and heterogeneous hyperintense to mildly hyperintense on T2-weighted imaging (T2WI). No definite enhancement was observed on post-contrast images, and the lesion appeared hypointense, closely adjacent to the pectoralis major muscle.

#### 2.1.4 Pathology from bard magnum core needle biopsy

The pathology confirmed IDC with ER (+80%), PR (+90%), HER2 (–), and Ki-67 (50%) expression. The findings were consistent with Luminal B-like BC, staged as pT2N0M0 according to the AJCC 8th edition.

### 2.2 Diagnostic and therapeutic management of the primary tumor

**Figure 1:**
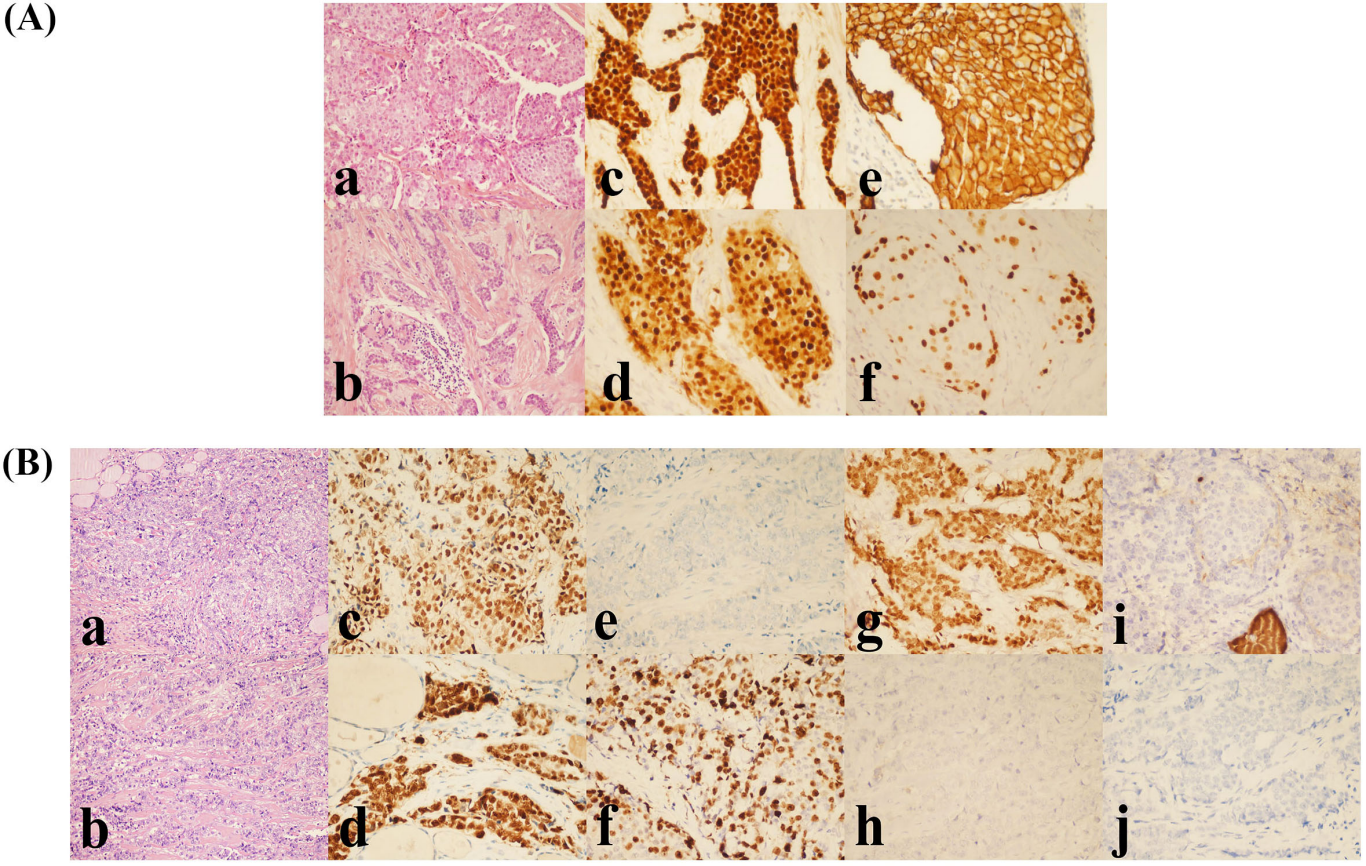
**(A)** Histopathological and IHC features of the primary breast tumor. **(a, b)** Grade 2 IDC (H&E, ×200). **(c)** Estrogen receptor (ER) positive (90%). **(d)** Progesterone receptor (PR) positive (80%). **(e)** HER2 IHC score 2+. **(f)** Ki-67 proliferation index (30%). All IHC panels **(c-f)** were visualized at ×400 magnification. **(B)** Histopathological and IHC features of MBCT. **(a, b)** Poorly differentiated carcinoma infiltrating the right thyroid lobe (H&E, ×200). **(c)** ER positive (80%). **(d)** PR positive (90%). **(e)** HER2 negative (score 0). **(f)** Ki-67 proliferation index (70%). **(g)** GATA3 positive. **(h)** PAX-8 negative. **(i)** Thyroglobulin (Tg) negative. **(j)** TTF-1 negative. All IHC panels **(c-j)** were visualized at ×400 magnification.

Considering the patient’s breast-conserving intent and tumor size, eight cycles of neoadjuvant chemotherapy (AC-T regimen) were administered from October 2015 to March 2016. Serial tumor measurements closely monitored the patient’s response during chemotherapy. The clinical response assessment indicated partial response (PR) according to RECIST criteria. Upon completion of preoperative evaluation, no absolute contraindications to surgery were identified. Subsequently, the patient underwent breast-conserving surgery with sentinel lymph node biopsy (SLNB) in April 2016. Postoperative pathology confirmed IDC, histological grade 2, with a residual tumor measuring 1.8 cm × 1.5 cm × 1.3 cm (ER + 90%, PR + 80%, HER2 2+, FISH-negative, Ki-67 30%) (**Figure 1A**). No lymphovascular or perineural invasion was identified, and all surgical margins (superior, inferior, medial, lateral, and deep) were free of carcinoma. Compared with the pre-chemotherapy biopsy specimen, postoperative pathology revealed a Miller-Payne grade 3 treatment response. Three sentinel lymph nodes (SLNs) harvested from the left axilla during surgery were negative for metastatic carcinoma on intraoperative frozen section examination. Postoperatively, the patient received adjuvant radiotherapy to the whole breast at a dose of 50 Gy delivered in 25 fractions, with a subsequent 16 Gy boost to the tumor bed (cumulative dose 66 Gy). The patient also underwent endocrine therapy for five years.

### 2.3 Follow-up and detection of thyroid lesion

**Figure 2:**
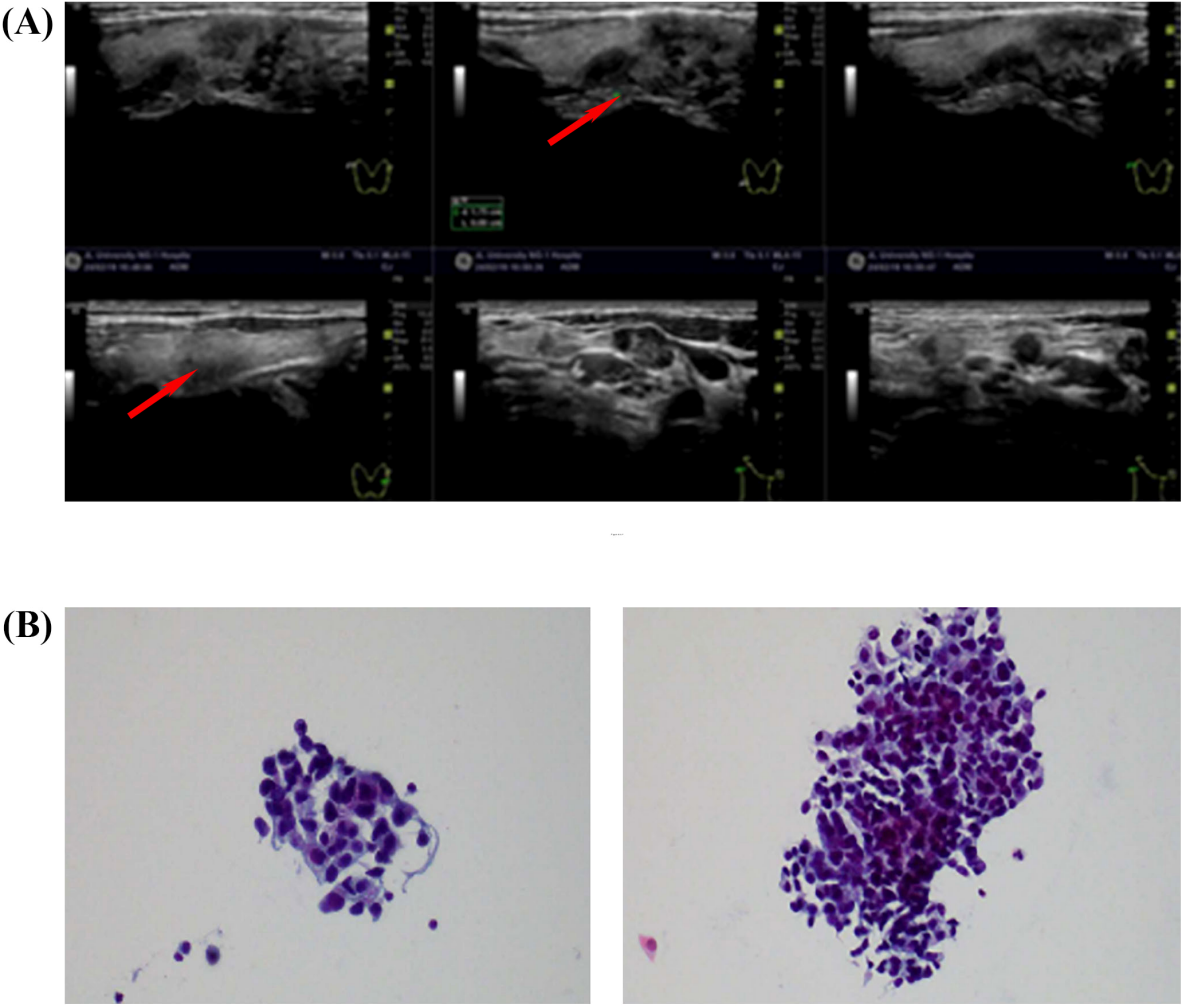
**(A)** Thyroid and regional lymph node color Doppler ultrasound (19 February 2024). **(B)** FNAC of thyroid and lymph node lesions. Smear from the right thyroid lobe (left). Aspirate from a right cervical level IV lymph node (right).

In February 2024, thyroid ultrasound examination revealed multiple nodules in the right thyroid lobe. The dominant nodule, located in the lower pole, measured 10 × 6.4 mm and presented as a solid hypoechoic lesion with ill-defined margins, an irregular shape, and no distinct capsule. Color Doppler flow imaging (CDFI) demonstrated punctate peripheral vascularity around the lesion. Additionally, patchy hypoechoic areas appeared in the right thyroid lobe. The largest lesion, located in the middle portion, measured 15 × 7.6 mm (transverse × anteroposterior), showing ill-defined margins, irregular contour, and heterogeneous internal echogenicity, with minimal vascularity on CDFI. Multiple hypoechoic lymph nodes appeared in the right cervical regions (levels II, III, IV, and V). The largest lymph node at level IV measured 13 × 7 mm and exhibited well-defined margins, irregular cortical contour, and partial fatty hilum effacement. The dominant thyroid nodule was categorized as C-TIRADS 4B (moderate malignancy suspicion), while other nodules were assigned C-TIRADS 3 (low suspicion) based on their overall sonographic presentation(**Figure 2A**). FNAC of the right thyroid lobe revealed atypical epithelial cells, suggesting possible papillary thyroid carcinoma (PTC). FNAC of the right level IV cervical lymph node identified metastatic carcinoma cells, morphologically suggestive of PTC (**Figure 2B**). The measurement of thyroglobulin in the aspirate fluid was 0.96 ng/ml, which is below the normal reference range.

### 2.4 Management of thyroid lesion and confirmation of metastatic thyroid carcinoma

In February 2024, the patient underwent total thyroidectomy with central compartment lymph node dissection (level VI) and right-sided functional neck dissection (levels II–V). Postoperative histopathology demonstrated metastatic poorly differentiated carcinoma infiltrating the right thyroid lobe. Microscopically, the carcinoma displayed multifocal growth and diffuse infiltration into thyroid parenchyma and perithyroidal fibrous connective tissue. Extensive lymphovascular invasion (LVI) and perineural invasion (PNI) were evident. Clinical history combined with IHC findings confirmed metastatic breast carcinoma. Histological examination also revealed a micro-PTC in the left thyroid lobe (diameter <1 mm), showing unencapsulated invasive growth confined within the thyroid capsule. Neither LVI nor PNI was detected. Background chronic lymphocytic thyroiditis was identified. Metastatic carcinoma was absent in perithyroidal lymph nodes (0/1). Notably, intravascular carcinoma emboli consistent with metastatic breast carcinoma appeared in small vessels within extracapsular fibroadipose tissue. Metastatic carcinoma was detected in: left central compartment (level VI: 3/3 lymph nodes), right central compartment (level VI: 2/2 lymph nodes, 2 tumor deposits), and right lateral cervical lymph nodes (levels II–V: 5/7 lymph nodes, 3 tumor deposits). Morphological and IHC profiles of metastatic lesions confirmed metastatic breast carcinoma. Immunohistochemistry results were as follows: Right thyroid: GATA3 (+), ER (>80%), PR (>90%), HER2 (-), Ki-67 (70%), Tg (-), TTF-1 (-), PAX-8 (-); Lymph nodes: TTF-1 (-) (**Figure 1B**). Following surgical recovery, the case was re-evaluated by the multidisciplinary team. Given the confirmation of metastatic breast carcinoma to the thyroid and regional lymph nodes, the patient was classified as having stage IV disease. The cornerstone of postoperative management was systemic therapy, tailored to the luminal B (HER2-negative) molecular subtype of the primary breast cancer. The patient was started on a regimen of an aromatase inhibitor combined with a CDK4/6 inhibitor. Additionally, a structured follow-up protocol was established, including regular clinical examinations and cross-sectional imaging to monitor treatment response and detect any new sites of metastasis.

### 2.5 Follow-up and subsequent treatment

At the most recent follow-up in June 2025, comprehensive imaging studies, including contrast-enhanced CT of the chest, abdomen, and pelvis, revealed no evidence of additional distant metastases. The patient is currently undergoing therapy with an aromatase inhibitor (exemestane) combined with a CDK4/6 inhibitor (abemaciclib). She adheres well to the treatment regimen, which has been well-tolerated to date without any significant adverse events, disease progression, or evidence of acquired resistance.

## 3 Discussion

The pathogenesis of MBCT remains incompletely understood. It likely arises from a complex interaction between intrinsic tumor cell properties (e.g., homing, dormancy, and reactivation) and the host organ microenvironment, aligning with the ‘seed and soil’ hypothesis (12–14). Two principal patterns have been proposed: (1) The “seed and soil” theory offers a conceptual framework for understanding how pre-existing thyroid disorders, such as thyroiditis, may establish a permissive microenvironment—through mechanisms including altered oxygen tension and iodine levels—that facilitates the metastatic seeding and colonization of tumor cells originating from distant sites (15). This theory provides a potential mechanistic explanation for MBCT. It is noteworthy, however, that given the high detection rate of incidental micro−PTC (16, 17), a cautious interpretation is warranted. The co−occurrence of these conditions should sometimes be regarded as a plausible association rather than as definitive evidence of causality. (2) The hematogenous dissemination concept suggests that TM from non-thyroid primaries predominantly utilize anatomical and physiological vascular pathways. In this scenario, circulating tumor cells (CTCs) extravasate into thyroid microvasculature and subsequently colonize the thyroid gland (18). In the present case, the patient had no personal or family history of thyroid disorders. However, postoperative pathology identified primary micropapillary carcinoma with focal lymphocytic thyroiditis in the left thyroid. This finding provides a possible mechanistic explanation for MBCT via microenvironmental remodeling, consistent with the ‘seed and soil’ theory. Although the thyroid gland is highly vascularized, it remains an uncommon metastatic site. Further research is needed to clarify whether this rarity results from effective local immune surveillance or other mechanisms.

The delayed occurrence of metastasis in this case—more than five years after initial diagnosis—is notable. This strongly suggests that tumor cells may have persisted in a dormant state within the thyroid microenvironment. Such dormant cells could later be reactivated by alterations in microenvironmental signals (e.g., inflammation), activation of tumor stem cells, or immune escape (19, 20). The regulatory mechanisms controlling tumor cell dormancy and reactivation in the thyroid gland represent a critical area for future investigation.

It should be emphasized that diagnosing MBCT poses significant clinical challenges. Metastatic thyroid carcinomas commonly present as asymptomatic thyroid nodules. Their sonographic appearance frequently resembles primary thyroid cancers, particularly PTC, and may even lack typical malignant features (21, 22). This similarity leads to diagnostic challenges and potentially inappropriate interventions, such as unnecessary total thyroidectomy or delayed systemic therapy for BC. FNAC often complicates diagnosis due to morphological overlap, including similar features such as acinar/trabecular patterns and solid sheets, often resulting in ambiguous cytological findings (10). The definitive identification of breast-derived metastatic carcinoma to the thyroid strongly depends on IHC staining. Metastatic breast carcinoma cells typically exhibit positive expression of mammary epithelial differentiation markers (e.g., GATA, Mammaglobin, ER/PR, TRPS1) and negative expression of thyroid follicular cell markers (e.g., Thyroglobulin, TTF-1, PAX-8) (10, 23–29). In patients with a BC history, clinicians should not overlook the possibility of thyroid metastasis originating from breast carcinoma. This study reviewed relevant literature published in the past decade. **Table 1** summarizes the clinicopathological characteristics and treatment approaches of selected representative cases (30–39). Currently, there is no standardized therapeutic protocol supported by high-level evidence due to the rarity of this condition. The selection and sequence of surgical resection, radiotherapy, and systemic therapy vary greatly between individuals, influenced primarily by the patient’s original BC molecular subtype, overall disease burden (including the presence or absence of DM), general physical condition, and whether thyroid lesions produce local symptoms (22, 30). Therefore, enhanced recognition of clinical features and diagnostic challenges associated with MBCT is critical for improving diagnostic accuracy and optimizing treatment management.

**Table 1:** Reported cases of breast cancer metastasis to the thyroid gland.

Study	Year	Sex	Age (years)	Histology	Molecular Subtype	Interval between Breast cancer and thyroid metastasis (months)	Others metastasis	Treatment	Survival time after diagnosis (months)
Matilde Pensabene et al. (25)	2018	Female	56	Invasive Lobular Carcinoma	Luminal A	6	Bone	Surgery	41
Kevin Bourcier et al. (26)	2018	Female	54	Invasive Lobular Carcinoma	NA	0	NA	Surgery, Endocrine therapy	NA
Yan-Yan Zhang et al. (27)	2020	Female	36	Invasive Ductal Carcinoma	HER2	28	Neck LN	Surgery	NA
Yichao Wang et al. (28)	2021	Female	54	Invasive Ductal Carcinoma	HER2	24	Axillary lymph nodes, Chest wall	Chemotherapy, Targeted therapy	NA
Yupei Yu et al. (29)	2021	Female	59	Invasive Micropapillary Carcinoma	Luminal A	60	Chest wall	Surgery, Chemotherapy, Targeted therapy	NA
Xue Bai et al. (30)	2023	Female	30	Invasive Ductal Carcinoma	HER2	62	Stomach, Pleural, Chest wall, Lung lymphangitis carcinomatosa, Pancreas, Adrenal	Palliative chemotherapy, Targeted therapy	11
Masae Hoshi et al. (31)	2023	Female	58	Invasive Ductal Carcinoma	Luminal B	156	Axillary lymph nodes, Supraclavicular lymph nodes	Surgery	NA
Wenjuan Meng et al. (32)	2024	Female	54	Invasive Ductal Carcinoma	TNBC	0	NA	Chemotherapy, Immunotherapy,	NA
Luis A Ramírez Stieben et al. (33)	2024	Female	56	Invasive Ductal Carcinoma	Luminal B (HER2+)	60	Lung, Lymphatic, Bone, Nervous system	Surgery, Radiotherapy, Chemotherapy	NA
Souheil Jbali et al. (34)	2025	Female	63	Invasive Lobular Carcinoma	Luminal B	NA	Neck LN	Surgery, Radiotherapy, Chemotherapy, Endocrine therapy	NA

This diagnostic process in this case exemplifies the complexity involved in diagnosing MBCT. During follow-up eight years after BC surgery, thyroid ultrasound revealed an abnormal echo in the right thyroid lobe, resembling primary PTC. FNAC suggested PTC. However, postoperative pathological examination identified carcinoma cells with morphological features consistent with BC origin. The literature consistently indicates that PTC is characterized by a distinct set of nuclear features, including nuclear grooves, intranuclear pseudoinclusions, nuclear membrane irregularity, and pale, glassy chromatin with characteristic clearing (so-called Orphan Annie eye nuclei). The cytoplasm is typically eosinophilic. In contrast, MBCT may demonstrate colorless intranuclear holes due to possible mechanical distortion. Follicular thyroid carcinoma (FTC) usually presents significant monomorphic cellular architecture and prominent microfollicular patterns, whereas MBCT commonly exhibits pleomorphic cells and lacks microfollicular structures (10, 35). Thus, if morphologically distinct tumor cells appear on FNAC, clinicians should consider metastatic carcinoma and perform IHC staining to confirm the diagnosis. In the present case, definitive diagnosis depended on systematic IHC examination of the postoperative specimen. The IHC profile, positive for breast-specific markers and negative for thyroid-associated markers, confirmed metastatic breast carcinoma rather than a primary thyroid tumor. The inability to establish a definitive preoperative diagnosis in this patient highlights a significant clinical consideration. For patients with a BC history—especially if remote—even when FNAC or imaging studies (including suspicious lymph nodes) suggest primary thyroid carcinoma, clinicians must maintain a high index of suspicion for MBCT. Definitive histopathological examination following surgery, particularly systematic IHC staining encompassing both breast-specific markers (GATA3, mammaglobin, ER/PR) and thyroid-specific markers (thyroglobulin, TTF-1, PAX8), constitutes the diagnostic gold standard. For patients unsuitable for surgery due to medical intolerance or with unresectable masses, or for whom preoperative diagnostic confirmation is necessary, systematic IHC testing on FNAC specimens is crucial to prevent misdiagnosis and inappropriate management.

## 4 Conclusion

This rare case of MBCT, with an eight-year latency period, underscores the necessity of maintaining a high index of suspicion for thyroid metastasis in breast cancer survivors who present with thyroid nodules, particularly since imaging and cytological findings can be indistinguishable from primary thyroid cancer. A definitive diagnosis is achieved through immunohistochemistry, which demonstrates positivity for breast markers (GATA3, ER, PR,TRPS1) and negativity for thyroid markers (TTF-1, Tg, PAX-8). Although surgery may be utilized for diagnostic confirmation or symptom management, tailored systemic therapy constitutes the cornerstone of treatment for metastatic disease, highlighting the critical importance of a combined local and systemic approach to optimize outcomes in such rare metastatic presentations.

## 5 Materials and methods

Retrospective collection and analysis of a case of MBCT was conducted. Written informed consent was obtained from the patient for the use of the patient’s clinical information for research purposes. A copy of the written consent is available for review upon request to the Editor-in-Chief of this journal. This study was approved by the Ethics Committee of Jilin University First Hospital (Approval Number: 2023-KS-321) and followed the revised guidelines of the Declaration of Helsinki (2013).

## Data Availability

The raw data supporting the conclusions of this article will be made available by the authors, without undue reservation.
